# Structure Characterization of *Escherichia coli* Pseudouridine Kinase PsuK

**DOI:** 10.3389/fmicb.2022.926099

**Published:** 2022-06-17

**Authors:** Xiaojia Li, Kangjie Li, Wenting Guo, Yan Wen, Chunyan Meng, Baixing Wu

**Affiliations:** ^1^Guangdong Provincial Key Laboratory of Malignant Tumor Epigenetics and Gene Regulation, Guangdong-Hong Kong Joint Laboratory for RNA Medicine, RNA Biomedical Institute, Medical Research Center, Sun Yat-sen Memorial Hospital, Sun Yat-sen University, Guangzhou, China; ^2^Department of Obstetrics and Gynecology, Sun Yat-sen Memorial Hospital, Sun Yat-sen University, Guangzhou, China; ^3^Department of Biopharmaceutical Technology, School of Life Sciences, Guangzhou University, Guangzhou, China; ^4^Breast Tumor Center, Sun Yat-sen Memorial Hospital, Sun Yat-sen University, Guangzhou, China

**Keywords:** crystal structrue, pseudouridine (Ψ), kinase, nucleoside, *N*^1^-methyl-pseudouridine

## Abstract

Pseudouridine (Ψ) is one of the most abundant RNA modifications in cellular RNAs that post-transcriptionally impact many aspects of RNA. However, the metabolic fate of modified RNA nucleotides has long been a question. A pseudouridine kinase (PsuK) and a pseudouridine monophosphate glycosylase (PsuG) in *Escherichia coli* were first characterized as involved in pseudouridine degradation by catalyzing the phosphorylation of pseudouridine to pseudouridine 5′-phosphate (ΨMP) and further hydrolyzing 5′-ΨMP to produce uracil and ribose 5′-phosphate. Recently, their homolog proteins in eukaryotes were also identified, which were named PUKI and PUMY in *Arabidopsis*. Here, we solved the crystal structures of apo-*Ec*PsuK and its binary complex with Ψ or *N*^1^-methyl-pseudouridine (m1Ψ). The structure of *Ec*PsuK showed a homodimer conformation assembled by its β-thumb region. *Ec*PsuK has an appropriate binding site with a series of hydrophilic and hydrophobic interactions for Ψ. Moreover, our complex structure of *Ec*PsuK-m1Ψ suggested the binding pocket has an appropriate capacity for m1Ψ. We also identified the monovalent ion-binding site and potential ATP-binding site. Our studies improved the understanding of the mechanism of Ψ turnover.

## Introduction

Post-transcriptional RNA modifications regulate various RNA species and influence gene expression (Barbieri and Kouzarides, 2020). More than 160 modifications in RNAs have been found until now (Boccaletto et al., 2018). Among them, *N*^6^-methylated adenine (m^6^A) and pseudouridine (Ψ) are the most prevalent naturally occurring modifications (Gilbert et al., 2016; Zaccara et al., 2019); furthermore, Ψ is also considered the first discovered RNA modification (Cohn and Volkin, 1951). As an isoform of uridine, Ψ has been detected in tRNAs, rRNAs, mRNAs, snoRNAs, and snRNAs existing in all three domains of life; therefore, Ψ is sometimes referred to as the fifth RNA nucleoside because of its ubiquitous nature (Zaringhalam and Papavasiliou, 2016; Lin et al., 2021). For instance, Ψs in tRNA molecules account for around 2-5% of all identified tRNA modifications (Lin et al., 2021). Conserved Ψ sites in rRNAs across different species are found to stabilize various local motifs (Sharma and Lafontaine, 2015). H/ACA box snoRNAs that mediate RNA pseudouridylation are known to carry Ψs themselves mostly in the regions involved in base pairing with target sites (Carlile et al., 2014). All snRNAs are predominantly modified with Ψs mainly located in the functionally important regions (Morais et al., 2021). Transcriptome-wide studies have also mapped many Ψ sites in mRNAs in yeast, human, and human pathogens (Carlile et al., 2014; Schwartz et al., 2014; Li et al., 2015; Nakamoto et al., 2017). In general, Ψs are considered to affect the RNA structure, further stabilize the structure of the functionally important areas, and tune ribosome functions for efficient and accurate protein translation (Schwartz et al., 2014). Interestingly, the incorporation of Ψ or its synthetic derivative *N*^1^-methyl-pseudouridine (m1Ψ) was validated to escape from degradation by ubiquitous RNases, significantly decreasing the immunogenic nature of mRNA vaccine and improving the antigen production (Kariko et al., 2005, 2008; Andries et al., 2015; Pardi et al., 2018).

RNA modifications are usually dynamically introduced and removed by specific enzymes (Zaccara et al., 2019). The formation of Ψ is catalyzed by the pseudouridine synthases (PUS), which can be further subcategorized into RNA guide-dependent and stand-alone enzyme modes (Ganot et al., 1997; Ni et al., 1997; Rintala-Dempsey and Kothe, 2017). The production of Ψ involves an isomerization process, in which the base reposition of uracil occurred by replacing the carbon–nitrogen glycosidic bond (C^1^–N^1^) with a carbon–carbon bond (C^1^–C^5^) (Veerareddygari et al., 2016; Motorin and Marchand, 2021), and then a free N^1^ position is exposed (Deb et al., 2019). The loss of Ψs by mutations in the pseudouridine synthases leads to several diseases, such as growth retardation (Han et al., 2015; Balogh et al., 2020), neuronal dysfunctions, behavior defects (de Brouwer et al., 2018), and Crohn’s disease (Festen et al., 2011).

**FIGURE 1:**
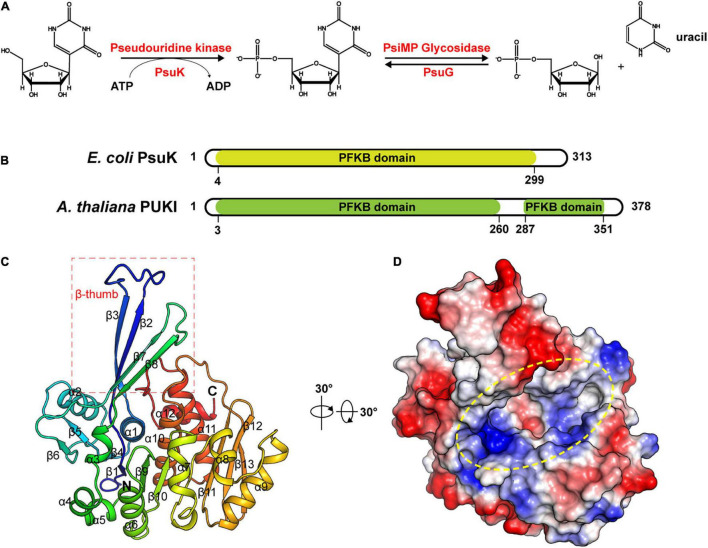
Structure of apo-*Ec*PsuK. **(A)** Reaction scheme of pseudouridine metabolism. **(B)** Domain organization of *Arabidopsis* PUKI and *E. coli* PsuK. **(C)** Overall structure of *Ec*PsuK is colored in the rainbow. β-Thumb region is indicated by a red box. **(D)** Surface representation of apo-*Ec*PsuK, and the potential substrate-binding pocket is indicated by a yellow oval.

Compared to the knowledge regarding the biogenesis and functions of RNA modifications, the metabolic fate of non-canonical nucleotides derived from the degradation of modified RNAs has just started. *N*^6^-methyl-adenosine monophosphate (*N*^6^-mAMP) produced from the metabolic turnover of m^6^A-containing RNAs was demonstrated to be degraded by an *N*^6^-mAMP-specific deaminase named ADAL that hydrolyzes *N*^6^-mAMP to inosine monophosphate (IMP), which is an intermediate of either purine nucleotide biosynthesis or catabolism in plants and human cells (Chen et al., 2018; Baccolini and Witte, 2019; Witte and Herde, 2020). Two subsequent structural studies validated the function of ADAL and identified the key residues in the active site that mediate the substrate specificity (Jia and Xie, 2019; Wu et al., 2019). Compared to *N*^6^-mAMP turnover, degradation of ΨMP was initially observed in the study with pyrimidine auxotrophic *Escherichia coli* mutants (Breitman, 1970). Detailed investigations of these mutants led to the discovery of YeiC and YeiN in *E. coli*, which were also known as pseudouridine kinase (PsuK) and pseudouridine-5′-phosphate glycosidase (PsuG) (Preumont et al., 2008), respectively. The catabolic process for Ψ consists of two steps: PsuK phosphorylates pseudouridine to 5′-ΨMP, and PsuG further hydrolyzes 5′-ΨMP, producing uracil and ribose 5′-phosphate (Figure 1A; Preumont et al., 2008; Chen and Witte, 2020). Recently, the homolog enzymes *At*PUKI and *At*PUMY in *Arabidopsis* were also described (Figure 1B; Chen and Witte, 2020). Like IMP, uracil may be reincorporated into uridine monophosphate in the salvage reaction or may enter pyrimidine ring catabolism (Loh et al., 2006; Zrenner et al., 2009). Malfunction of these catabolic enzymes was validated to be toxic and causes delayed seed germination and growth inhibition (Chen and Witte, 2020).

Intriguingly, PsuK and PsuG are present in many organisms from bacteria to eukaryotes (Preumont et al., 2008; Chen and Witte, 2020), whereas in metazoa, amoebozoa, and fungi, homologs of PsuG and PsuK reside on a single polypeptide chain, representing eukaryotic ΨMP glycosylase physically linked to pseudouridine kinase. By contrast, mammals generally lack these enzymes, except for platypus (*Ornithorhynchus anatinus*) (Chen and Witte, 2020). Previous biochemical and structural observations for *At*PUKI showed its high specificity toward pseudouridine (Chen and Witte, 2020; Kim et al., 2021) and illustrated how *At*PUKI discriminates pseudouridine from other structurally similar pyrimidine nucleosides or derivates. Nevertheless, although *Ec*PsuK and its homolog protein *At*PUKI both belong to the phosphofructokinase B (PfkB) family of carbohydrate kinases, they share a low sequence identity of about 21% (Park and Gupta, 2008; Chen and Witte, 2020). The specific catalytic mechanism for *Ec*PsuK to Ψ has just begun to be uncovered; a residue Ser30 was suggested to play a key role in promoting the catalytic reaction by inducing the conformational change in this specific kinase (Kim et al., 2022).

In this study, we determined crystal structures of *E. coli* PsuK in apo-form and its binary complex with Ψ or m1Ψ. Our results provide a structural rationale for the high preference of *Ec*PsuK for the non-canonical nucleoside pseudouridine and *N*^1^-methyl-pseudouridine. Furthermore, the undiscovered side effect of Ψ-containing RNAs appears strikingly advantageous for the development of generations of mRNA-based vaccines. Our studies put forward a hypothesis for the nucleoside-modified mRNA vaccine degradation pathway.

## Results

### Structure of Apo-*Ec*PsuK

To unveil the catalytic mechanism of *Ec*PsuK for the pseudouridine substrate, we first obtained the crystal structure of apo-*Ec*PsuK; the crystal of apo-*Ec*PsuK was determined at 2.3Å which belonged to the space group *P*6_3_22. Detailed diffraction statistics can be found in Table 1. There is one *Ec*PsuK molecule in the asymmetric unit, which can be modeled from Arg2 to Asn308 and folded into a conformation employing the β-α unit as a basic structural motif composed of a central α/β region, and a β-stranded region protruding from the N-terminal (Figure 1C). In detail, the interlaced β-α units in the α/β domain prompt the formation of a central β-sheet, which contains eight β-strands that are positioned in the order of β6-β5(β4)-β1-β9-β10-β11-β12-β13 with a parallel orientation, except for β12; seven α-helices (α3-α9) were further arranged on one side of the central β-sheet and the remaining α-helices (α1, α2, α10–α12) on the other side. Furthermore, two β-strands β2 and β3 between the β1–α1, β7, and β8 between the β6–α3 extend from the central α/β structure region. These two consecutive β-strands are corporately constituting the antiparallel β-sheet in an edge-to-edge orientation (hereafter named β-thumb region) (Figure 1C). These structural conformations of *Ec*PsuK present a groove alongside the β-thumb region above the central β-sheet that is full of charged residues (Figure 1D).

**TABLE 1:** Data collection and refinement statistics.

	Apo-*Ec*PsuK (7VKP)	*Ec*PsuK-Ψ (7VSK)	*Ec*PsuK-m1Ψ(7W93)
**Data collection**			
Wavelength	0.97915	0.97853	0.97915
Space group	*P*6_3_22	*P*6_3_22	*P*6_3_22
**Cell dimension**			
*a*, *b*, *c* (Å)	186.18, 186.18, 52.21	182.634, 182.634, 51.228	185.83, 185.83, 52.51
α, β, γ (°)	90, 90, 120	90, 90, 120	90, 90, 120
Resolution (Å)	30.0–2.30 (2.38–2.30)*	30.0–2.30 (2.38–2.30)	30.0–1.90 (2.00–1.90)
*R*_merge_	0.148 (0.520)	0.137 (0.454)	0.146 (1.462)
*I*/σ*I*	22.25 (3.5)	13.67 (3.0)	15.8 (2.2)
Completeness (%)	99.9 (99.9)	99.5 (98.2)	98.5 (97.1)
Redundancy	21.0 (9.6)	17.7 (8.3)	28.2 (21.6)
**Refinement**			
Resolution (Å)	30.0–2.30	30.0–2.30	30.0–1.90
No. reflections	22,978	21,673	41,719
*R*_work_/*R*_free_	0.219/0.249	0.228/0.275	0.196/0.215
No. atoms	2,373	2,140	2,532
Protein	2,321	2,059	2,386
Ligand/Water	52	81	146
*B*-factors	42.82	40.03	51.26
**R.m.s. deviations**			
Bond lengths (Å)	0.008	0.008	0.009
Bond angles (°)	0.939	0.988	1.098
**Ramachandran plot**			
Favored/allowed (%)	95.74/4.26	95.15/4.85	96.72/3.28

*Highest resolution shell is given within parentheses.

### Dimeric Structure of *Ec*PsuK

**FIGURE 2:**
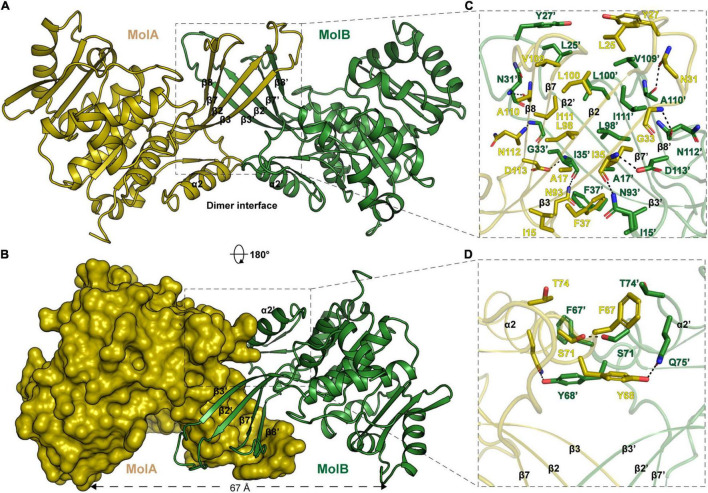
Structure of *Ec*PsuK homodimer. **(A)** Dimeric structure of *Ec*PsuK is shown as the cartoon; the two protomers are colored yellow and green. The dimer interface is indicated by a black box. **(B)** Surface representation of the dimer interface. **(C)** Detailed interactions for dimerization within the β-thumb region. The hydrogen bonds are shown as black dashed lines. **(D)** Detailed interactions between α2 and α2′. The hydrogen bonds are shown as black dashed lines.

The calculated molecular weight of monomeric *Ec*PsuK (aa. 1–313) is 33.6 kDa, but the purified protein had a molecular weight of ∼67 kDa determined by size exclusion chromatography (SEC), indicating that it is a dimer in solution (Supplementary Figure 1A). Consistently, our structure also showed that *Ec*PsuK was a dimer when we analyzed the adjacent asymmetric unit (Figure 2A). Therefore, although there is only one *Ec*PsuK molecule in the asymmetric unit, it is a homodimer. Dimerization of *Ec*PsuK is mainly assembled by two regions including the β-thumb region and α2 on the edge of the central β-sheet (Figures 2A,B). The dimer interface adopts a face-to-face mode that looks like a butterfly with the four β-strands in the β-thumb region almost perpendicular to these elements from the adjacent asymmetric unit, which created a cross β-barrel-like fold (Figure 2A; Sigrell et al., 1998).

In detail, the β-thumb region of each monomer of the *Ec*PsuK homodimer rigidly contacts with each other through many hydrophobic interactions, creating a hydrophobic core (Figure 2C). The residues involved in the contacts contain Ile15, Ala17, Leu25, Tyr27, Asn31, Gly33, Ile35, Phe37, Leu98, Leu100, Val109, Ala110, and Ile111 with a face-to-face mode to their counterparts from another molecule. Hydrophilic interactions are also observed in this β-barrel-like region, the side chain of Asn31 forms a H-bond with the main chain of Ala110′, and the main chain of Ile35 is synchronously hydrogen-bonded to the side chain of Asp113′ and Asn93′ (Figure 2C). In the α2-α2′ region, two aromatic residues Phe67 and Tyr68 in the center account for the hydrophobic interactions together with Thr74; meanwhile, the hydroxyl group of Tyr68 interacts with the side chain of Gln75′ *via* a hydrogen bond, and the side chain of serine interacts with its counterparts of the other molecule (Figure 2D). Taken together, these interactions anchored the *Ec*PsuK homodimer.

### The Binary Complex of *Ec*PsuK-Ψ

**FIGURE 3:**
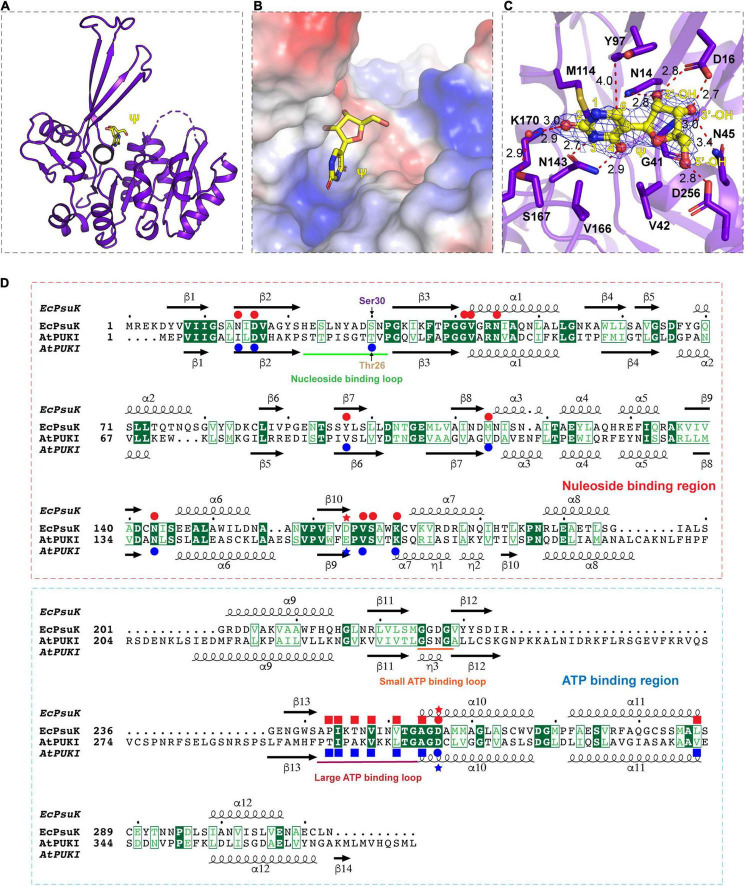
Complex structure of *Ec*PsuK with Ψ. **(A)** Overall structure of *Ec*PsuK in complex with Ψ. *Ec*PsuK is shown as cartoon and colored in purple, and Ψ is shown as the stick and colored in yellow. **(B)** Substrate-binding surface underneath the β-thumb region and above the α/β fold region. **(C)** Detailed interactions between *Ec*PsuK and Ψ. *Ec*PsuK is shown as cartoon and colored in purple, the residues in contact with Ψ are shown as sticks, and Ψ is shown as stick colored in yellow. The hydrogen bonds are shown as red dashed lines. The red sphere represents the water molecule. The 2| Fo| –| Fc| σ-weighted map is contoured at 1.5σ. **(D)** Structure-based sequence alignment between *At*PUKI and *Ec*PsuK. The residues involved in substrate binding are indicated by red (*Ec*PsuK) or blue (*At*PUKI) circles, respectively. The residues involved in ATP binding are indicated by blue squares for *At*PUKI, and the red squares indicated the potential residues in *Ec*PsuK involved in ATP binding. The nucleoside-binding region and the ATP-binding region are indicated by red and blue rectangles, respectively. The red and blue stars indicate the residues that may involve in catalytic reaction.

To further illustrate the substrate binding properties of *Ec*PsuK with pseudouridine, we determined the crystal structure of *Ec*PsuK complexed with pseudouridine through co-crystallization, and further soaking of the crystals with both pseudouridine and ADP. We successfully obtained the complex structure, which also belongs to the space group *P*6_3_22, and diffracted it to 2.30 Å (Table 1). However, we can only achieve electron density for pseudouridine, and the ADP cannot be modeled (Figure 3A); thus, we named the structure of the binary complex *Ec*PsuK-Ψ. The superposition of both the monomeric and dimeric structures of the *Ec*PsuK-Ψ complex with apo-*Ec*PsuK revealed minor conformational differences (r.m.s.d about 0.295 Å) (Supplementary Figure 1B). However, a loop region (Ile244 to Gly253) and the C-terminal (Met285 to Asn308) are lost in this binary structure, which is discussed later.

In the *Ec*PsuK-Ψ complex, pseudouridine is well accommodated at the cleft alongside the β-thumb domain, with the nucleobase inserted into the pocket and the 5′-OH of the ribose group points to the orientation of the unoccupied region in the cleft (Figure 3B). The base of pseudouridine is well recognized by a number of hydrophobic and hydrophilic interactions with good densities (Figures 3C,D and Supplementary Figure 1C). For instance, the O^2^ position hydrogen binds to the side chain of K170 and the side chain of Ser167 mediated by a water molecule, respectively. The N^3^ and O^4^ positions are bound to the side chain of Asn143. The base is also well clamped by the side chain of Met114, Val166, and Tyr97, which forms a hydrophobic environment with the nucleobase of pseudouridine. The ribose group is also well anchored by hydrogen contacts from Asn14, Asp16, and Asn45 (Figures 3C,D). The 2′-OH forms hydrogen binds with the side chain NH2 group of Asn14 and the side chain of Asp16; similarly, the 3′-OH of pseudouridine is hydrogen-bonded to the side chain Asp16, main chain amino group of Gly41, and the side chain of Asn45. The 5′-OH position forms a hydrogen bond with the side chain of Asp256, which is considered one of the residues involved in catalyzing the reaction (Figure 3D, indicated by asterisks) (Kang et al., 2019; Kim et al., 2021). Furthermore, Gly41 and Val42 hydrophobically interact with the ribose ring to stabilize the conformation of pseudouridine. It is interesting to find that the residues involved in the recognition of the pseudouridine are mainly located in the N-terminal part of the *Ec*PsuK end to α7, except for the Asp256 (Figure 3D), and the following region is a loop between α7 and α8 with good density; moreover, these residues are very conserved in *At*PUKI (Figure 3D; Kim et al., 2021). Taken together, the structure of the *Ec*PsuK-Ψ binary complex suggested that pseudouridine is specifically recognized by *Ec*PsuK mainly by the N-terminal half.

### Structure Comparisons of *Ec*PsuK With *At*PUKI

**FIGURE 4:**
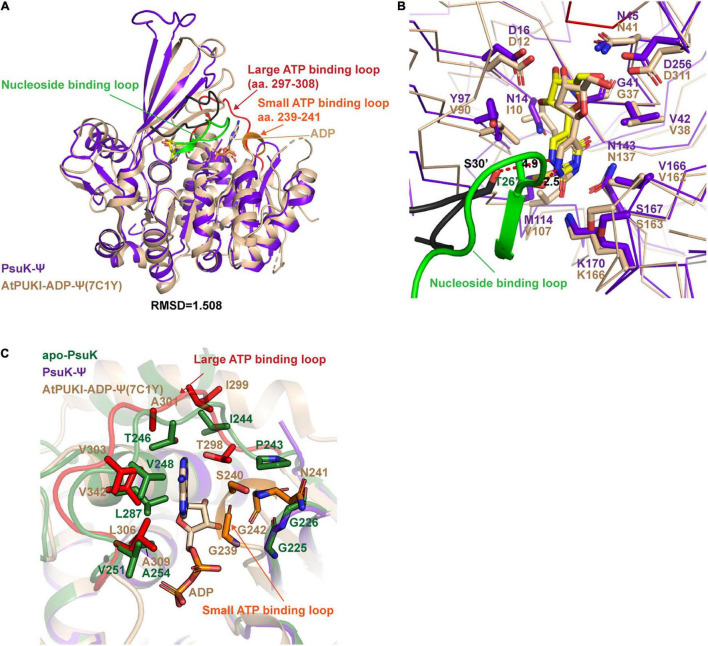
Structure comparisons between *Ec*PsuK and *At*PUKI. **(A)** Structure superposition of the *Ec*PsuK-Ψ complex with the *At*PUKI-ADP-Ψ complex (PDB code: 7C1Y). The nucleoside-binding loop, ATP-binding loop, and ADP in *At*PUKI are all indicated by arrows. **(B)** Comparisons of the substrate-binding pocket between *Ec*PsuK and *At*PUKI. The nucleoside-binding loop containing T26′ is colored green. The residues involved in substrate recognition are shown as stick. The black-colored loop is the corresponding nucleoside-binding loop in *Ec*PsuK, and the residue S30’ is colored in black and shown as stick. **(C)** Detailed comparisons of the ATP-binding site between *Ec*PsuK and *At*PUKI. The residues used for binding ADP in *At*PUKI, and the corresponding residues in *Ec*PsuK are all shown as stick.

To further dissect the catalytic mechanism of *Ec*PsuK to the substrate, we compare the structures of apo-*Ec*PsuK and *Ec*PsuK-Ψ with the *At*PUKI-Ψ-ADP complex (PDB code:7C1Y) (Kim et al., 2021). Although *At*PUKI has an extra 65 residues length longer than *Ec*PsuK (Figure 1B), which has 313 residues, the superposition of monomeric apo-*Ec*PsuK to *At*PUKI monomer showed quite a minor difference with r.m.s.d about 1.263 Å (Supplementary Figure 2A). There are two insertions in *At*PUKI when compared with *Ec*PsuK; they are the loop region between α8 and α9 and a long-disordered region between β12 and β13 (Figure 3D). However, according to the complex structure of *At*PUKI-Ψ-ADP, the homodimeric structure of *At*PUKI adopts a transition mode to catalyze the reaction and leads to the differences in each protomer of *At*PUKI (Kim et al., 2021). Consistent with these observations, the superposition is quite different between dimeric *Ec*PsuK and *At*PUKI, when one molecule is superimposed well, and the other one has a high deviation (Supplementary Figure 2B). We then superimposed the structure of apo-*Ec*PsuK with another protomer of *At*PUKI, and the results showed that the r.m.s.d is 1.988 Å with the β-thumb region of *At*PUKI closer to the central α/β region, which is also observed when we compared the complex structure of *Ec*PsuK-Ψ with the same protomer of *At*PUKI (Figure 4A and Supplementary Figures 2C,D). This set of *At*PUKI can represent the dimeric structure status of the apo-state because the overall conformation of all the solved *At*PUKI structures has the same dimeric structure, and these two molecules represent the different catalytic-associated states (Supplementary Figure 2E) (Kim et al., 2021).

We further compared the structure of *Ec*PsuK-Ψ with the active state of the *At*PUKI-Ψ-ADP complex in detail, which showed an r.m.s.d. about 1.508 Å (Figure 4A). The substrate cleft in the *At*PUKI-Ψ-ADP complex contains a Ψ and an ADP molecule. A comparison of the pseudouridine recognition revealed that almost all the residues involved in specific interactions of the pseudouridine are well conserved with *Ec*PsuK, although with some substitution such as Val90 in *At*PUKI but a Tyr97 in *Ec*PsuK; Ile10 and Val107 in *At*PUKI are corresponding to Asn14 and Met114 of *Ec*PsuK, respectively; these substitutions will not impact the substrate binding properties (Figures 3D, 4B). Importantly, a key residue Thr26’ located in the so-called nucleoside-binding loop in the dimeric *At*PUKI plays a key role in recognizing the pseudouridine because the side chain hydroxyl group of Thr26’ can form a 2.5 Å hydrogen bond with the N^1^ position of pseudouridine (Kim et al., 2021), whereas in *Ec*PsuK, it is a Ser30’ with 4.9 Å to the N^1^ position (Figure 4B). Previous studies showed that the T26S mutant of *At*PUKI has similar Km and kcat values for the pseudouridine, like that of the wild-type *At*PUKI (Kim et al., 2021). These analyses suggested that the Ser30’ in *Ec*PsuK has the potential to bind the N^1^ position of pseudouridine and further improve the specificity of the substrate.

The ADP is bound by two so-called ATP-binding loops including the large ATP-binding loop and the small ATP-binding loop in *At*PUKI (Kim et al., 2021) (Figures 3D, 4C). However, in our binary complex structure of *Ec*PsuK-Ψ, the large ATP-binding loops have been lost and without any electron density of ADP (Figure 3A). The two regions, the loop (Ile244 to Gly253) and the C-terminal (Met285 to Asn308) of *Ec*PsuK, are corresponding to the region wherein *At*PUKI was observed to bind to the monovalent ion. Although we failed to obtain the complex structure of *Ec*PsuK with ADP, we compared the ATP-binding pocket of *Ec*PsuK to *At*PUKI with the apo-*Ec*PsuK structure. Combined with sequence alignment, the ATP-binding region is also conserved within the large ATP-binding loop full of hydrophobic residues to stack contact with the nucleobase, and the sequence of the small ATP-binding loop showed a conserved “GXXG” motif (Figures 3D, 4C). Whereas the small ATP-binding loop in *At*PUKI has a much more rigid conformation for the nucleobase of ATP accommodation than that in *Ec*PsuK, these differences may result from the existence of ADP, which pushes the “GXXG” motif closer to the nucleobase in *At*PUKI (Figure 4C). Compared to the pseudouridine-binding site, the residues involved in ATP binding are all located in the C-terminal part of *Ec*PsuK; thus, the α/β domain may also be divided into two parts in *Ec*PsuK and *At*PUKI, which can be defined as the nucleotide-binding region and ATP-binding region (Figure 3D).

### The Complex Structure of *Ec*PsuK With *N*^1^-Methyl-Pseudouridine (m1Ψ)

During the crystallization process of the *Ec*PsuK-Ψ complex, we used the gel filtration buffer containing 10mM Tris pH 8.0 and 100mM NaCl to purify the *Ec*PsuK protein. The disorder of the loop region (Ile244 to Gly253) and the C-terminal (Met285 to Asn308) may be attributed to the weak interactions of sodium with *Ec*PsuK for anchoring these regions in the presence of ADP. Therefore, we changed the gel filtration buffer to 10mM Tris pH 8.0 and 100mM KCl, which contains the substituted monovalent ion potassium to purify the *Ec*PsuK protein. Furthermore, compared to the Ser26’ in *At*PUKI structures that are directly involved in the binding to the N^1^ position of pseudouridine (Kim et al., 2021), Ser30’ in *Ec*PsuK is much far from the N^1^ position of pseudouridine observed in our *Ec*PsuK-Ψ complex (Figure 4B). Despite it may impact the transition status for catalytic reaction, we aimed to understand if *Ec*PsuK has the binding ability to N^1^-substituted pseudouridine, one such example is m1Ψ, in which the hydrogen of the N^1^ position of pseudouridine is substituted by a methyl group. Previous studies tested many nucleotide analogs of Ψ such as 5-methyl uridine (Chen and Witte, 2020; Kim et al., 2021); however, there is still no evidence of the binding ability of *Ec*PsuK to Ψ derivates.

**FIGURE 5:**
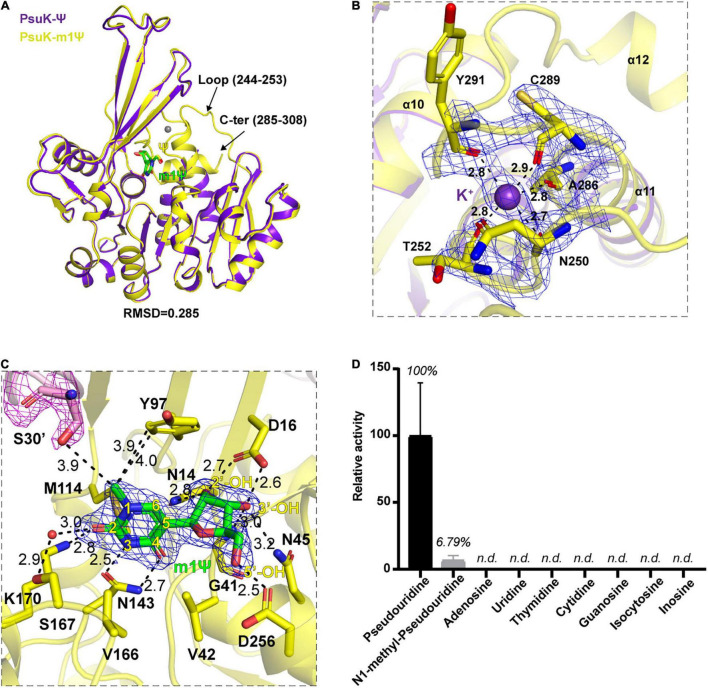
Structure complex of *Ec*PsuK with m1Ψ. **(A)** Structure superposition of *Ec*PsuK-m1Ψ and *Ec*PsuK-Ψ. m1Ψ is colored in green, and Ψ is colored in yellow. The lost regions in the *Ec*PsuK-Ψ complex are indicated by black arrows. **(B)** Binding site of a monovalent ion. The 2| Fo| –| Fc| σ-weighted map is contoured at 1.5σ. K^+^ is colored purple shown as a sphere. **(C)** Detailed interactions between *Ec*PsuK and m1Ψ. *Ec*PsuK is shown as cartoon and colored in yellow, the residues in contact with Ψ are shown as sticks, and m1Ψ is shown as stick colored in green. The hydrogen bonds are shown as black dashed lines. The red sphere represents the water molecule. The 2| Fo| –| Fc| σ-weighted map is contoured at 1.5σ. **(D)** Substrate specificity of *Ec*PsuK for pseudouridine and other nucleoside analogs. n.d., not detected.

To test this hypothesis, we then co-crystallized *Ec*PsuK with m1Ψ in the presence of potassium and ADP. We then obtained the complex structure of *Ec*PsuK with m1Ψ with higher resolution at 1.90 Å (Table 1); however, the density of ADP still could not be found in this complex structure. Compared to the *Ec*PsuK-Ψ complex with an r.m.s.d only 0.285, in this *Ec*PsuK-m1Ψ structure, the lost regions can be well modeled (Figure 5A). The density of K^+^ can be achieved due to the high resolution and bound by the main chain of surrounding residues including Asn250, Thr252, Ala286, Cys289, and Tyr291 (Figure 5B). We further analyzed the binding environment of m1Ψ with good densities (Supplementary Figure 2F), and the comparisons between Ψ and m1Ψ in the substrate-binding pocket revealed that the interactions between *Ec*PsuK and m1Ψ are a little more stringent than those in *Ec*PsuK-Ψ (Figures 3C, 5C). Without major conformational changes, Tyr97 donates more hydrophobic interactions with the *N*^1^-methyl group in m1Ψ, and the pairing of Lys170 and Asn143 with m1Ψ is more intensive than that with Ψ (Figure 5C, Supplementary Figures 1C,2E). Meanwhile, the side chain hydroxyl group showed a 3.9 Å distance from the *N*^1^-methyl group (Figure 5C). These observations suggested that *Ec*PsuK has the binding capacity for the substrate of *N*^1^-substituted pseudouridine.

We then compared the catalytic activity of *Ec*PsuK to Ψ, m1Ψ, and many other nucleoside analogs by a direct activity assay method (Andersson and Mowbray, 2002). The assay results showed that *Ec*PsuK kept a weak activity of about 6.79% toward the m1Ψ substrate relative to the Ψ substrate; nevertheless, *Ec*PsuK revealed no catalytic activity to the other nucleoside analogs including adenosine, uridine, thymidine, cytidine, guanosine, isocytosine, and inosine (Figure 5D). Taken together, these results suggested that although m1Ψ can be bound by *Ec*PsuK just as our structure showed, it is not the most suitable substrate for *Ec*PsuK.

### Structural Homologs of *Ec*PsuK

**FIGURE 6:**
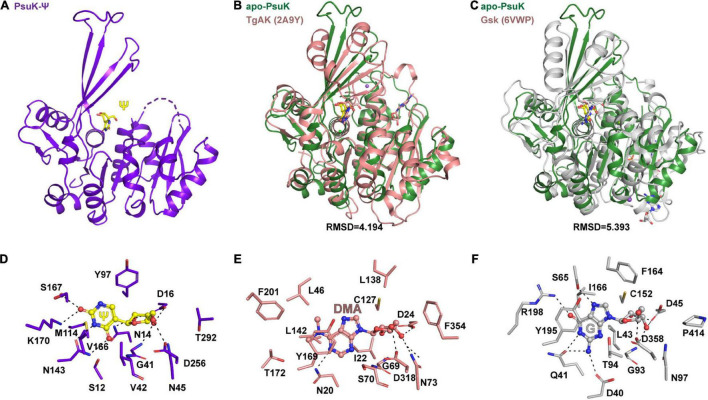
Comparisons of substrate binding properties of *Ec*PsuK with TgAK and Gsk. **(A)** The overall structure of *Ec*PsuK in complex with Ψ. **(B)** Structure superposition of *Ec*PsuK-Ψ with TgAK-DMA complex (PDB code: 2A9Y). The latter complex structure is colored in pink. **(C)** Structure superposition of *Ec*PsuK-Ψ with Gsk-guanosine complex (PDB code: 6VWP). The latter complex structure is colored in white. **(D)** Detailed interactions between PusK with Ψ. **(E)** Detailed interactions between TgAK with DMA (*N*^6^, *N*^6^-dimethyladenosine). **(F)** Detailed interactions between Gsk with guanosine.

*Ec*PsuK is a member of the PfkB family (Park and Gupta, 2008); accordingly, structural features of monomeric and dimeric *Ec*PsuK are also highly homologous to those of this protein family. A structure similarity search using the program DALI (Holm, 2020) indicated that the *Ec*PsuK monomer exhibits high structural homology with the PfkB family kinases catalyzing the phosphorylation of ribose. Based on the DALI search results, we selected the published structures with nucleoside kinase activity to further analyze the similarities and differences among the nucleoside kinase of the PfkB family (Table 2). They are two known function nucleoside kinases containing the adenosine kinase from *Toxoplasma gondii* (*Tg*AK, PDB code: 2A9Y) and inosine–guanosine kinase from *E. coli* K12 (Gsk, PDB: 6VWP) (Figures 6A–C). DALI results revealed that the *Ec*PsuK and *Tg*AK share only 17% sequence identity, but with the structural homology of about 4.194 Å, and the *Ec*PsuK and *Ec*Gsk share about 20% sequence identity with 5.393 Å (Figures 6B,C and Supplementary Figure 3). The *Tg*AK presents as a monomeric conformation that contains a nucleotide-binding pocket essentially at an equivalent location to where it is found in *Ec*PsuK and *At*PUKI (Figure 4A). Compared with *Ec*PsuK, the pocket of *Tg*AK is larger to allow the accommodation of a nucleoside with a purine base (Figures 6D,E). In the complex structure of *Tg*AK with *N^6^,N^6^*-dimethyladenosine (DMA), the nucleobase-binding part of the pocket lacks hydrophilic residues that could mediate specific hydrogen bonds to the adenine moiety (Figure 6E). By contrast, guanosine–inosine kinase (Gsk) protein can recognize guanine with high specificity, in which the nucleobase is specifically bound *via* many hydrophilic interactions (Figure 6F).

**TABLE 2:** Information of the *Ec*PsuK homolog proteins.

No.	PDB	Z-score	rmsd	%id	Description	References
1	7c1y	37.4	2.9	21	PSEUDOURIDINE KINASE	Kim et al., 2021
2	2a9y	31.4	2.6	17	ADENOSINE KINASE	Zhang et al., 2007
3	6vwp	27.3	2.9	20	INOSINE-GUANOSINE KINASE	Wang et al., 2020

### Conserved Substrate-Binding Site in the Eukaryotic PsuG-PsuK Fusion Proteins

In some eukaryotic organisms, the putative enzymes responsible for the catabolism of pseudouridine are physically fused to a polypeptide (Chen and Witte, 2020). We then performed a multiple sequence alignment with *At*PUKI, *At*PUMY, *Ec*PsuK, *Ec*PsuG, and their potential homolog proteins in yeast (*Schizosaccharomyces pombe*), zebrafish (*Danio rerio*), fly (*Drosophila melanogaster*), and nematode (*Caenorhabditis elegans*). However, all these enzymes we selected have not been defined and were without a formal name; thus, we named these bifunctional enzymes pseudouridine kinase glycosidase (PsuKG) following the nomenclature of PsuK and PsuG in *E. coli*. The N-terminal of the PsuKG proteins represents PsuG, which is very conserved in these species mentioned earlier (Supplementary Figure 4). In the C-terminal half, about 350 residues are well-conserved putative pseudouridine kinases (Supplementary Figure 4). We performed a structure-based sequence analysis, whose structures are derived from the solved structure in *E. coli*, plants, and the structures predicted by Alphafold2 (Supplementary Figures 5A–H) (Jumper et al., 2021). Based on our observations, the residues that participate in the recognition of pseudouridine are all well conserved, except in flies, in which the key residue Lys170 pairing with the nucleobase of pseudouridine in *E. coli* is substituted with isoleucine (Supplementary Figures 4, 5E). The residue for N^1^ position recognition is either a serine or threonine in all the protein sequences we showed, indicating the transition reaction scheme for pseudouridine may be necessary and conserved during evolution (Supplementary Figure 4). For the ATP-binding region, the small ATP-binding loop is GXXG in yeast and zebrafish, but a serine in flies and alanine in the nematode (Supplementary Figures 4, 5B,D,F,H). The large ATP-binding loop is much more conserved and is full of aromatic residues. These comparisons indicated the potential catabolic function of the PsuKG proteins for pseudouridine. Whether or not these proteins metabolize pseudouridine and also use ATP as the phosphate group donor needs further investigation.

## Discussion

Pseudouridine is a type of widespread naturally occurred modification existing in almost every type of RNA. It was demonstrated that pseudouridine modification in RNAs can regulate many aspects of RNA fate such as RNA stability, translation efficiency, and base-pairing properties due to its additional N^1^ position compared with uridine. Although the catalytic installation mechanism has been well studied, the metabolism of modified nucleotides including pseudouridine has just started to be uncovered. Previous studies revealed that N^6^-mAMP was catalyzed by a specific deaminase ADAL and produced inosine, which can be further utilized by the purine salvage pathway (Chen et al., 2018; Jia and Xie, 2019; Wu et al., 2019). The enzyme that can recognize *N*^6^-methyl-adenosine was also determined not long ago (Jiang et al., 2021).

Recently, the metabolic pathway of pseudouridine in plants has been uncovered; two enzymes named *At*PUKI and *At*PUMY can sequentially phosphorylate the Ψ to ΨMP and hydrolyze 5′-ΨMP to produce uracil and ribose 5′-phosphate (Chen and Witte, 2020). This study in plants confirmed the initial finding in *E. coli*, in which the study showed that *Ec*PsuK and *Ec*PsuG have a similar function in metabolizing pseudouridine as *At*PUKI and *At*PUMY (Preumont et al., 2008). Here, we determined the structure of *E. coli* PsuK in a complex with Ψ or m1Ψ, and our studies revealed that the overall structure of *Ec*PsuK adopts a homodimer conformation with a face-to-face mode through the interaction of the β-thumb region and α2 with their counterparts (Figure 2A), and the monomeric *Ec*PsuK-Ψ showed high structural similarity with the inactive status of *At*PUKI (Supplementary Figure 2A). Ψ was captured by the α/β region mainly located in the N-terminal part of *Ec*PsuK alongside the β-thumb region. We identified the key residues in recognizing the pseudouridine substrate, and these residues are well conserved in *Ec*PsuK homolog proteins from different species (Supplementary Figure 4). We also obtained the complex structure of *Ec*PsuK with m1Ψ; comparisons of the binding properties between *Ec*PsuK-m1Ψ and *Ec*PsuK-Ψ suggested that *Ec*PsuK has binding capacity for an additional *N*^1^-methyl substituent group with weak catalytic activity.

Although we solved these complex structures, there are still many questions to be clarified. (1) We attempted to capture ATP or ADP, but no density could be observed in all the diffraction data we collected. The ATP-binding loop between *Ec*PsuK and *At*PUKI has some differences, especially in the small ATP-binding loop. Whether the binding of ATP can induce the conformational change in the small ATP-binding loop is still a question. (2) We have observed some extra density around the residues including Asp164, Glu190, Asn187, and Asp256; however, due to the lack of a phosphate group, we cannot model the bivalent metal ions because of the existence of some water molecules. Given that there is magnesium chloride in the crystallization conditions, we believed that the extra densities around these residues contain two Mg^2+^ ions; furthermore, all the crystals grown in the conditions lacking Mg^2+^ showed weak diffraction quality. These observations were consistent with the results shown in previous studies that the active site contains two Mg^2+^ in *At*PUKI (Kim et al., 2021). (3) We have attempted to change the crystallization conditions to rule out the crystal packing effect, but in all conditions, the crystals of apo-*Ec*PsuK or its complex belong to the *P*6_3_22 space group. Therefore, m1Ψ in our crystal structures seems more suitable in the substrate-binding pocket excluding the impact of Ser30’ from another molecule in the homodimer when in the inactive form. A similar study was published online when we prepared our manuscript (Kim et al., 2022); the transition status was also observed in that study. In their *Ec*PUKI-Ψ complex structure, there are eight molecules that can be considered four homodimers (Supplementary Figure 6A). Intriguingly, only one molecule of each homodimer contains the Ψ substrate; comparisons of these homodimer structures suggested a dynamic sensing mechanism with the active protomers bound to the substrate assistant by the bent β-thumb regions, and the other protomers without substrate were much more flexible (Supplementary Figure 6B). We analyzed the dimerization status in these homodimer structures in detail and found that the distance of Ser30’ with the N^1^-position presents a variable distance of about 4.6 Å in the Mol12 homodimer, 5.1 Å in the Mol34 homodimer, 3.3 Å and 3.9 Å in Mol56 and Mol78 homodimer structures, respectively (Supplementary Figures 6C–F). By contrast, our structure of either apo-*Ec*PsuK or *Ec*PsuK-(m1)Ψ complex showed more rigid homodimer conformation due to the crystallographic symmetry than the multiple structure status of *Ec*PUKI (Supplementary Figures 6G–J). Furthermore, *Ec*PUKI was suggested to bind the uridine and cytidine with both of the two protomers containing substrates in their binding pockets; however, these structures together with the structures of apo-*Ec*PUKI and *Ec*PUKI-S30A-Ψ showed the inactive form belong to the space group P3. Structure comparisons of our *Ec*PsuK-m1Ψ with these *Ec*PUKI structures revealed minor RMSD, these results validated that our *Ec*PsuK structures are in the inactive form (Supplementary Figures 6K–N), and this form leads to the omissive identification of the important function of Ser30’ in inducing the conformational change for catalysis. Therefore, although our studies demonstrated that the m1Ψ substrate could be bound comfortably in our *Ec*PsuK-m1Ψ complex in the inactive status, the methyl group of m1Ψ will impact the phosphorylation effectiveness. It is worth noting that all the dimers of *At*PUKI presented a transition status even in the unliganded *At*PUKI structure (Supplementary Figure 2E). (4) Last but not least, previous structure studies suggested that the PsuG in *E. coli* was a trimer (Huang et al., 2012), and *Ec*PsuK is a homodimer; the real tertiary and quaternary structure arrangement of the fusion protein PsuKG is still unknown.

mRNA vaccines have been demonstrated as a highly effective technique to cope with the COVID-19 pandemic; this successful experience promotes mRNA-based technologies as a promising method in cell therapies (Baden et al., 2021; Thomas et al., 2021). However, two major issues including the widespread degradation of exogenous RNA by ubiquitous RNases and the immunogenic nature of exogenous RNA need to be overcome while making use of the mRNA-based technologies. The incorporation of naturally occurring RNA modifications is an effective method to avoid these undesirable results (Kariko et al., 2005; Lockhart et al., 2019). Among these RNA modifications, fully replacing uridines with Ψ has been demonstrated to be a robust method that can enhance the stability of the parent mRNA and lead to strongly increased protein expression compared to the unmodified mRNAs (Kariko et al., 2008, 2011). Furthermore, incorporation of the synthetic derivative *N*^1^-methyl-pseudouridine can further improve translation efficiency and evade innate immune response (Andries et al., 2015; Svitkin et al., 2017). The hypermodified m1Ψ is formed *via* further methylation of Ψ. At present, at least five types of Ψ hypermodification, including Ψm and m1Ψ, are found in all domains of life (Spenkuch et al., 2014). For m1Ψ, a specific RNA methyltransferase Nep1 was demonstrated to responsible for the N^1^-specific Ψ methylation in the small ribosomal subunit RNA (Leulliot et al., 2008; Taylor et al., 2008; Wurm et al., 2010; Meyer et al., 2011). Mja_1640 from *M. jannaschii* was also validated to catalyze the N^1^-methylation of position Ψ54 located in the T-arm of tRNAs *in vitro* with the proper sequence specificity (Wurm et al., 2012). Mutation in human Nep1 results in a fatal developmental disorder known as Bowen-Conradi syndrome (Armistead et al., 2009). However, the specific demethylase of m1Ψ is still unknown, and the metabolic fate of these modified nucleotides does not attract much notice. PsuK and PsuG are present in organisms from bacteria to eukaryotes (Preumont et al., 2008; Chen and Witte, 2020), although in metazoa, amoebozoa, and fungi, these two proteins are fused to a single polypeptide chain. To our knowledge, there is still no homolog proteins of PsuK found in mammals, and how the pseudouridine-modified nucleosides of mRNA vaccine are degraded is still a question.

## Materials and Methods

### Protein Expression and Purification

Plasmids encoding *E. coli* PsuK (Uniprot ID: A0A140N873) were PCR amplified from *E. coli* BL21 (DE3) genome. The PCR product was double-digested with restriction endonuclease *BamH*I and *Xho*I and then ligated into a modified pET-28a plasmid carrying the Ulp1 cleavage site. Recombinant plasmids were confirmed by DNA sequencing and transformed into *Escherichia coli* BL21 (DE3) to produce target proteins with N-terminal His_6_-sumo fusions. *E. coli* cells were cultured in the LB medium at 37°C with 50 mg/L kanamycin until the OD_600_ reached 0.6–0.8, then the bacteria were induced with 0.2 mM isopropyl-β-d-thiogalactoside (IPTG) at 18°C for 16 h. Bacteria were collected by centrifugation; resuspended in buffer containing 20 mM Tris–HCl pH 8.0, 500 mM NaCl, 20 mM imidazole pH 8.0; and lysed by high pressure. Cell extracts were centrifuged at 18,000 rpm for 1 h at 4°C. Supernatants were purified with Ni-NTA (GE), the target protein was washed with lysis buffer, and then eluted with a buffer containing 20 mM Tris–HCl, pH 8.0, 500 mM NaCl, and 500 mM imidazole. Ulp1 protease was added to remove the N-terminal tag and fusion protein of the recombinant protein and dialyzed with lysis buffer for 3 h. The mixture was applied to another Ni-NTA resin to remove the protease and uncleaved proteins. Eluted proteins were concentrated by centrifugal ultrafiltration, loaded onto a pre-equilibrated HiLoad 16/60 Superdex 200-pg column, and eluted at a flow rate of 1 ml/min with the buffer containing 10 mM Tris–HCl pH8.0, 100 mM NaCl or 10 mM Tris–HCl pH8.0, 100 mM KCl. Peak fractions were analyzed by SDS-PAGE (15%, w/v) and stained with Coomassie Brilliant Blue R-250. Purified fractions were pooled together and concentrated by centrifugal ultrafiltration. The protein was concentrated at 10 mg/ml for crystallization trials determined by A_280_.

### Crystallization and Data Collection

Apo-*Ec*PsuK was crystallized using the hanging drop vapor diffusion method by mixing 1 μl of protein and 1 μL of reservoir solution at 18°C. The crystal suitable for X-ray diffraction was grown in a reservoir solution consisting of 0.02 M magnesium chloride hexahydrate, 0.1 M HEPES pH 7.5, 22% w/v poly (acrylic acid sodium salt) 5,100 (Hampton Research). For the *Ec*PsuK-Ψ complex, the crystals suitable for data collection were first co-crystallized of *Ec*PsuK with ADP and Ψ in the reservoir solution containing 20% (w/v) polyacrylic acid 5,100, 0.1 M HEPES/sodium hydroxide pH 7.0, 0.02 M magnesium chloride, and were further soaked with cryoprotectant containing the solution supplied with 25% glycerol, 1mM Ψ and 4mM ADP. For the *Ec*PsuK-m1Ψ complex, the crystals suitable for data collection were first co-crystallized of *Ec*PsuK with ADP and m1Ψ in the reservoir solution containing 20% (w/v) polyacrylic acid 5,100, 0.1M HEPES/sodium hydroxide pH 7.0, and 0.02M magnesium chloride and were further soaked with a cryoprotectant containing the solution supplied with 25% glycerol, 1mM m1Ψ, and 4mM ADP.

### Structure Determination and Refinement

For the apo-*Ec*PsuK structure, the diffraction data set was processed and scaled using HKL3000 or imosflm (Minor et al., 2006; Battye et al., 2011). The phase was determined by molecular replacement using the program PHASER with the structure of *At*PUKI (PDB code: 7C1Y) as the search model (McCoy et al., 2007). Cycles of refinement and model building were carried out using REFMAC5 and COOT, respectively (Emsley and Cowtan, 2004; Murshudov et al., 2011). For the *Ec*PsuK-Ψ and *Ec*PsuK-m1Ψ complex, the phase was determined by molecular replacement using the PHASER program with apo-*Ec*PsuK as the search model. The details of data collection and processing are presented in Table 1. All structure figures were prepared with PyMOL.

### Activity Assay

A direct assay was utilized to measure *Ec*PsuK activity to the nucleoside substrates (Andersson and Mowbray, 2002), as reported previously for the assays of *At*PUKI and *Ec*PUKI (Kim et al., 2021, 2022). In this direct assay, the reaction mixture contained 40 mM Tris–HCl pH 7.5, 20 mM MgCl_2_, 50 mM KCl, 0.003% phenol red, 4 mM ATP, and 200 nM wild-type *Ec*PsuK. The mixture was incubated at 25°C for 2 min, then the absorbance was monitored using the Thermo Evolution 201 at 430 nm. To trigger the enzyme reaction, 1.25 mM pseudouridine or other nucleosides were added to this mixture, and the absorbance at 430 nm was monitored again after 30 s. The substrates used for testing the activity contain pseudouridine, N^1^-methyl-pseudouridine, adenosine, uridine, thymidine, cytidine, guanosine, isocytosine, and inosine. All assays were conducted in triplicate.

## Data Availability Statement

The datasets presented in this study can be found in online repositories. The names of the repository/repositories and accession number(s) can be found in the article/Supplementary Material.

## Author Contributions

BW conceived the project, solved the structures and interpreted the experimental data. XL and KL expressed, purified, and grew crystals of the *Ec*PsuK. BW, YW, WG, CM, and XL collected X-ray diffraction data. BW and XL wrote and revised the manuscript. All authors contributed to the article and approved the submitted version.

## Conflict of Interest

The authors declare that the research was conducted in the absence of any commercial or financial relationships that could be construed as a potential conflict of interest.

## Publisher’s Note

All claims expressed in this article are solely those of the authors and do not necessarily represent those of their affiliated organizations, or those of the publisher, the editors and the reviewers. Any product that may be evaluated in this article, or claim that may be made by its manufacturer, is not guaranteed or endorsed by the publisher.
