# Supplementary material for: Structure Characterization of Escherichia coli Pseudouridine Kinase PsuK
Source: Front Microbiol. 2022 Jun 17;13:926099. doi: 10.3389/fmicb.2022.926099 (PMC9247573; doi:10.3389/fmicb.2022.926099)
Supplement: Supplementary file 1 [file Data_Sheet_1.PDF]

Supporting Information For

Structure characterization of *E. coli* pseudouridine kinase PsuK

Xiaojia Li[1,2,#], Kangjie Li[4, #], Wenting Guo[1], Yan Wen[1,3], Chunyan Meng[1], Baix ing Wu[1,*]

[1] Guangdong Provincial Key Laboratory of Malignant Tumor Epigenetics and Gene Regulation, Guangdong-Hong Kong Joint Laboratory for RNA Medicine, RNA Biomedical Institute, Medical Research Center, Sun Yat-Sen Memorial Hospital, Sun Yat-Sen University, Guangzhou, China 510120.

[2] Department of Obstetrics and Gynecology, Sun Yat-Sen Memorial Hospital, Sun Yat-Sen University, Guangzhou 510120, Guangdong, China

[3] Breast Tumor Center, Sun Yat-Sen Memorial Hospital, Sun Yat-Sen University, Guangzhou, 510120, China.

[4] School of Life Sciences, Guangzhou University, Guangzhou, 510120, China.

[*] Co-corresponding authors: Baixing Wu (E-mail: wubx28@mail.sysu.edu.cn)

[#] The authors wish it to be known that, in their opinion, the first two authors should be regarded as joint First Authors.

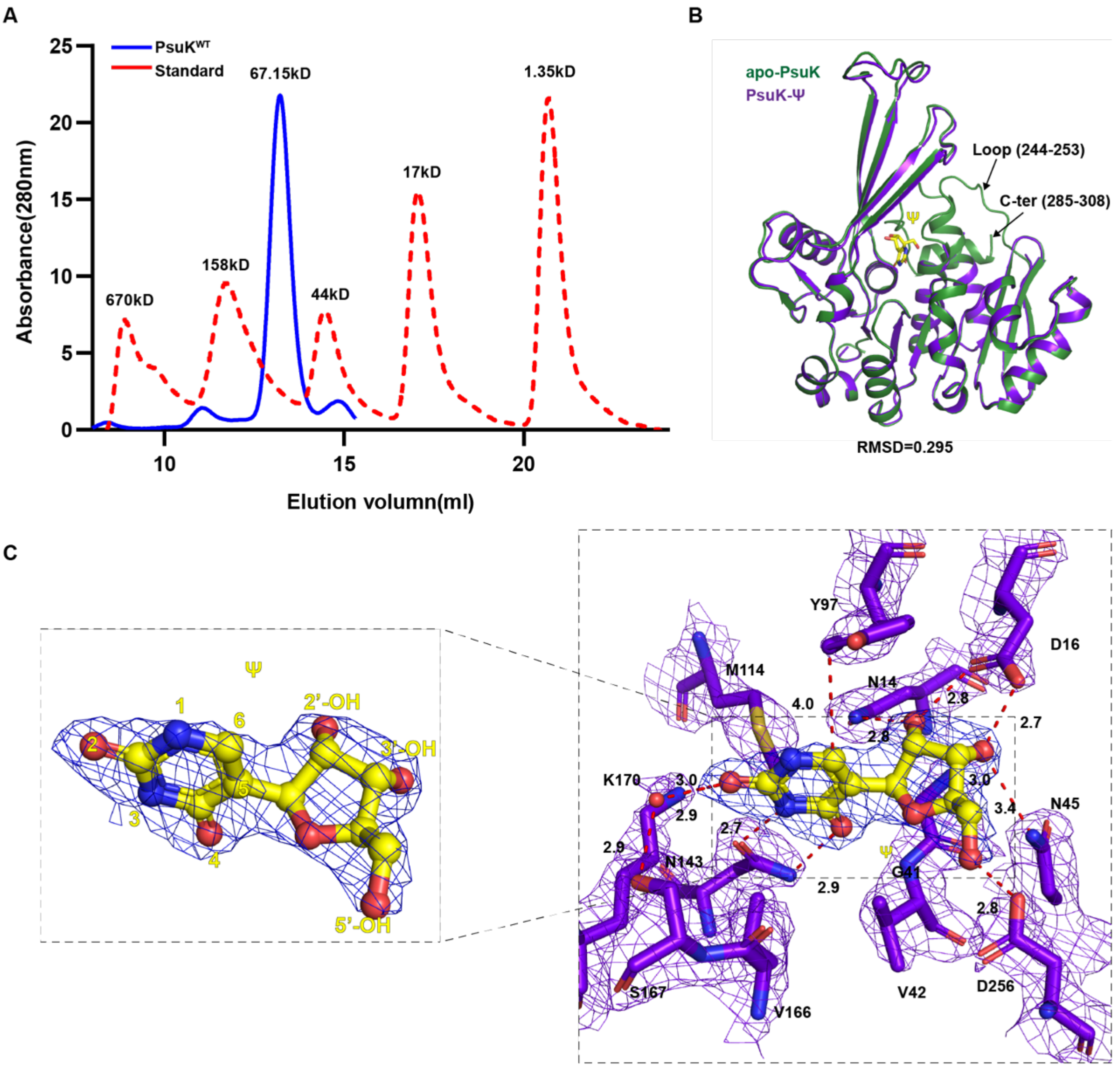

Supplementary Figure 1 The structure of *E. coli Ec*PsuK is a homodimer. (**A**) In-solution validation of the aggregation status of the *Ec*PsuK by gel filtration analysis. The protein standard is shown as red lines. The *Ec*PsuK protein is shown as blue line. (**B**) Superposition of the *Ec*PsuK-Ψ complex with apo-*Ec*PsuK. Apo-*Ec*PsuK is colored in teal and the *Ec*PsuK-Ψ complex is colored in purple-blue. The lost regions in the *Ec*PsuK-Ψ complex are indicated by black arrows. The Ψ is colored in yellow and shown as stick. (**C**) The residues in contact with Ψ are all shown as sticks. The red sphere represents the water molecule. The 2|Fo|–|Fc| σ-weighted map is contoured at 1.5σ. The map of the residues involved in contacting substrate is colored in purple, and the map of Ψ is colored in blue. The interactions are indicated by red dashed lines.

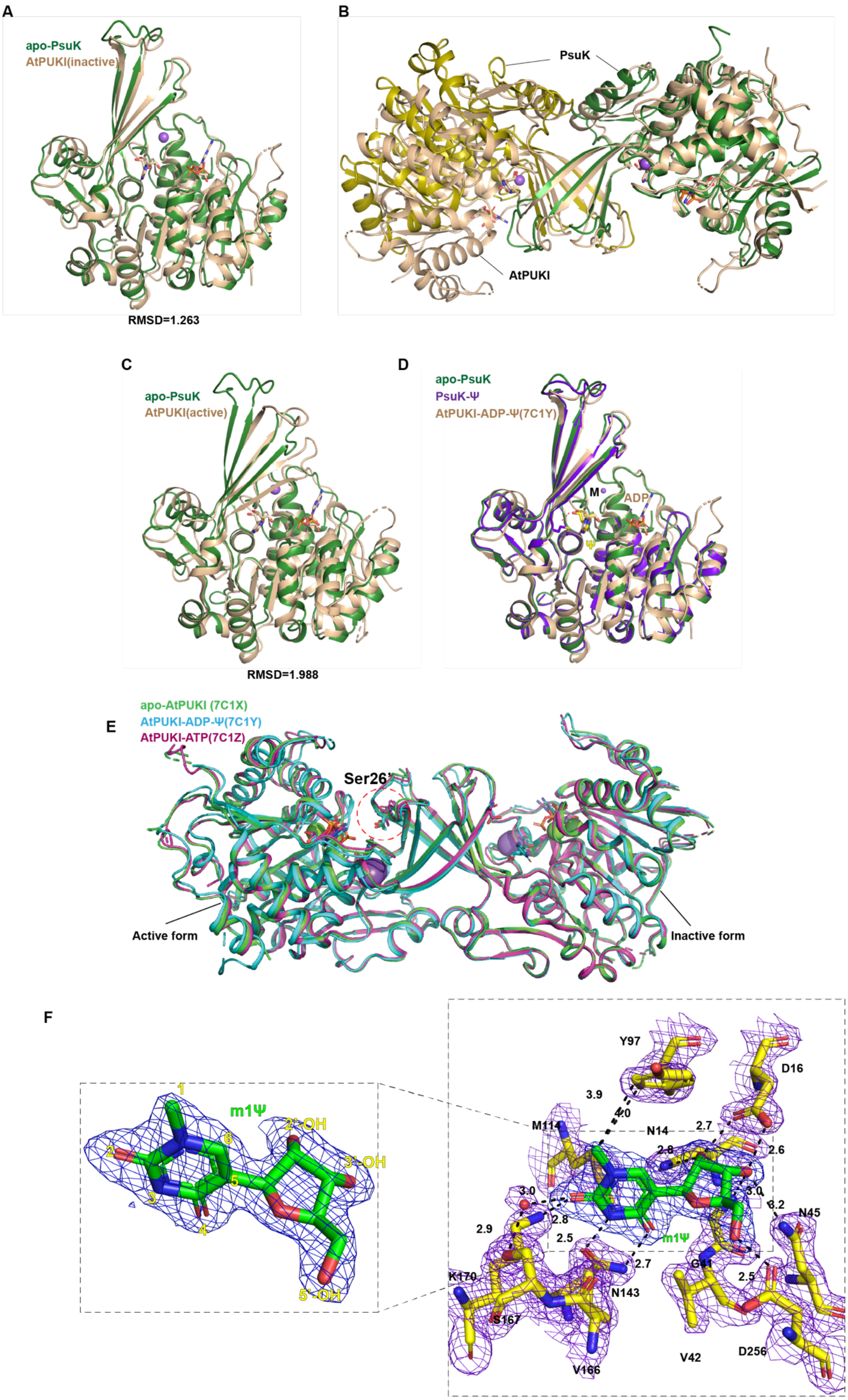
A
apo-PsuK
AtPUKI(inactive)
RMSD=1.263
B
PsuK
AtPUKI
C
apo-PsuK
AtPUKI(active)
RMSD=1.988
D
apo-PsuK
PsuK-Ψ
AtPUKI-ADP-Ψ(7C1Y)
M
ADP
Ψ
E
apo-AtPUKI (7C1X)
AtPUKI-ADP-Ψ(7C1Y)
AtPUKI-ATP(7C1Z)
Active form
Inactive form
F
m1Ψ
1
2
3
4
5
6
2'-OH
3'-OH
5'-OH
Y97
D16
3.9
M114
N14
2.7
2.8
2.6
3.0
3.0
3.2
N45
2.9
2.8
2.5
2.7
G41
2.5
K170
N143
S167
V166
V42
D256

Supplementary Figure 2 Structure of *Ec*PsuK presents an inactive state compared with *At*PUKI. (**A**) Superposition of monomeric *Ec*PsuK with *At*PUKI (inactive form). The monovalent ion in *At*PUKI is colored in purple and shown as a sphere. The substrate is shown as stick. The structure of *At*PUKI is colored in wheat, and the *Ec*PsuK is colored in teal. (**B**) Superposition of dimeric *Ec*PsuK with dimeric *At*PUKI. The dimeric structure of *At*PUKI is colored in wheat. The two protomers of the dimeric *Ec*PsuK-Ψ complex are colored in teal and yellow, respectively. (**C**) Superposition of monomeric *Ec*PsuK with *At*PUKI (active form). (**D**) Superposition of monomeric *Ec*PsuK and *Ec*PsuK-Ψ complex with *At*PUKI-ADP-Ψ complex (inactive form). The monovalent ion in *At*PUKI is colored in purple shown as sphere. The Ψ in the *Ec*PsuK-Ψ complex is shown as stick colored by yellow, and the ADP and Ψ in the *At*PUKI-ADP-Ψ complex are shown as stick colored wheat. (**E**) The structure comparisons of apo-*At*PUKI (PDB: 7C1X) with *At*PUKI-ADP-Ψ (7C1Y) and *At*PUKI-ATP(7C1Z). The Ser26' in *At*PUKI is circled by red dashed line. (**F**) The residues in contact with m1Ψ are all shown as sticks. The red sphere represents the water molecule. The 2|Fo|–|Fc| σ-weighted map is contoured at 1.5σ. The map of the residues involved in contacting substrate is colored in purple, and the map of Ψ is colored in blue. The interactions are indicated by black dashed lines.

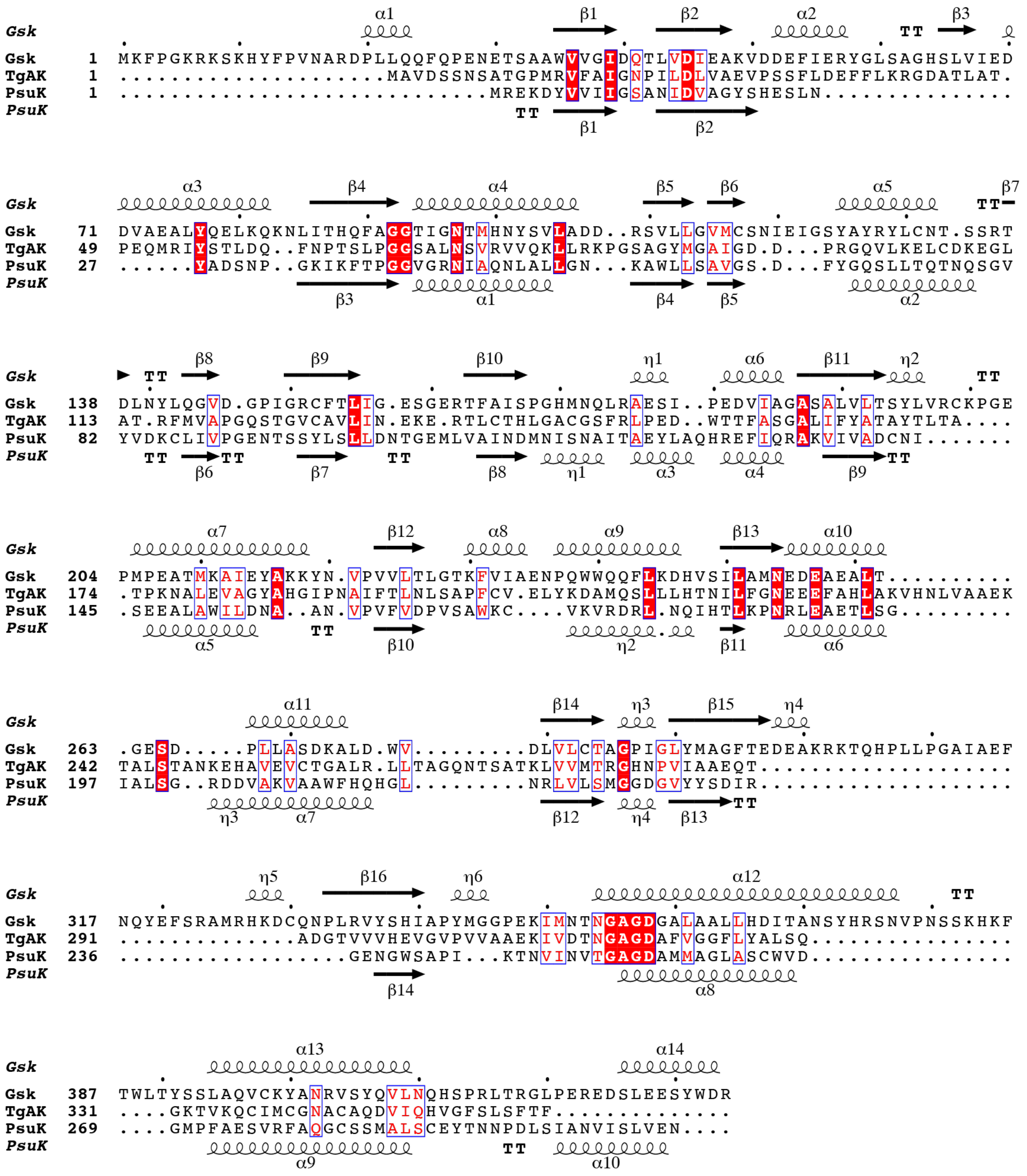

Supplementary Figure 3 Sequence alignment of *Ec*PsuK with TgAK and Gsk.

```
SpPsuKG   MLIVM---NRGCRLVSRIPLGFRRLKRNPNPGLRNILC-------RRYSPLALSKEVTEALKNNVPVVALESTIITHGMP   70
DrPsuKG   MLWRFSTFLRRCFTTT-----------------HSRHC-------KQDSLFTIHPDVKEALAHHRPVVALESTIITHGMP   56
DmPsuKG   MLLRRCF-LFPFPAVRLVR--------------RSLH--------RKHDHIVLHPEIRQALQLQKPVVALESTIITHGMP   57
CePsuKG   MCSRFF----------------------------GQKR-------SFSKFLQISPQVKEALASGEGVVALESTVITHGLP   45
EcPsuG    MSELK----------------------------------------ISPELLQISPEVQDALKNKKPVVALESTIISHGMP   40
AtPUMY    M----ASSLAQSRISN-----------------LQNHLSPLEANNKLRSLVKISPQVSEALSNGRAVVALESTIISHGMP   59
AtPUKI    --------------------------------------------------------------------------------
EcPsuK    --------------------------------------------------------------------------------

SpPsuKG   YPQNEELAIQVESKVRSMGAVPATIALLNGQCTIGLEQFQLSELAKSGET-AYKVSRRDLSYVASQRLNGGTTVAATMIL   149
DrPsuKG   YPHNLSTAKEVEAIVRAEGSIPATIGILEGRIHVGLSSDELDFLAQ-SKT-ALKVSRRDLPYVISKGLSGGTTVSGTMIA   134
DmPsuKG   MPENVVTALAVEEQVRQNGAIPATIGILDGRIKVGLTREELTSLAEKPRDQVIKCSRRDLPFVVSRRQSGGTTVAATMII   137
CePsuKG   YPHNLSTARSLEQKVRSSGSHPATIALFDGKIHVGLDDEKLELLAS-SQN-AVKVSSRDIAKTLIKKEVGGTTVASTMKI   123
EcPsuG    FPQNAQTAIEVEETIRKQGAVPATIAIIGGVMKVGLSKEEIELLGREGHN-VTKVSRRDLPFVVAAGKNGATTVASTMII   119
AtPUMY    YPQNLQTAKEVESIVRENGAIPATIAILNGVPCIGLSEEELERLASLGKS-VQKTAGRDIANVVATRGNGATTVSATLFF   138
AtPUKI    --------------------------------------------------------------------------------
EcPsuK    --------------------------------------------------------------------------------

SpPsuKG   ARAAGIDVFATGGIGGVHRGAENSMDISADLIELGRTRVAVVSAGVKSILDIGRTLEVLETQGVPVVTLGPP-KSAFPAF   228
DrPsuKG   ANKAGIPVFVTGGIGGVHRDGENSLDVSADLTELGRTPIAVVSAGVKSILDIGRTLEYLETQGVCVATYGDS--KSFPAF   212
DmPsuKG   AHRVGIHVFATGGIGGVHRDGHESMDVSADLTELGRTPVAVVCSGVKSILDIPRTLEFLETQGVCVASF-DSPGGVFPDF   216
CePsuKG   AHAAGISVFATGGIGGVHRGADQTFDVSADLQELSQTPVCVVCSGVKSILDIPKTVEYLETHSVNCIVYGQE--NVFPSF   201
EcPsuG    AALAGIKVFATGGIGGVHRGAEHTFDISADLQELANTNVTVVCAGAASILDLGLTTEYLETFGVPLIGYQT---KALPAF   196
AtPUMY    ASMVGIQVFVTGGIGGVHRHANHSMDISSDLTALGRTPIAVISAGVKSILDIPKTLEYLETQEVYVAAYKS---DEFPAF   215
AtPUKI    --------------------------------------------------------------------------------
EcPsuK    --------------------------------------------------------------------------------

SpPsuKG   FSRESKFQSPLSLETPQLIANMLFSNIQLGQECGTLVAIPTPHHCSIDYEKMEALIETCLQRSVQLGITGKNVTPWLLGE   308
DrPsuKG   FSRQSGFTSPYNISSPQEAADLIASTFSLGLKSGLLLAVPIPEEHAATGQQIEDAIQTAVTEASSKGVTGRDVTPFILQR   292
DmPsuKG   YTRDSGCTVPYNLKSAQEAAELLRSWRELKMESGLVIGVPIPEEFAADKFKIEEAIKEATAQARAQGISGKEVTPFLLAA   296
CePsuKG   FTRTSDRKAQFCTESLEEVVHLLKTSKSLGLPYGTILACPIPEKYAADGDAIQKAIDQAVQEAIEQNIASQSVTPFILAR   281
EcPsuG    FCRTSPFDVSIRLDSASEIARAMVVKWQSGLNGGLVVANPIPEQFAMPEHTINAAIDQAVAEAEAQGVIGKESTPFLLAR   276
AtPUMY    FTEKSGCKAPSRVNSPEDCARVIDANMKLNRQAGILFAIPIPKHHSAAGNLIESATQRALTEAREQNVTGNAETPFLLAR   295
AtPUKI    --------------------------------------------------------------------------------
EcPsuK    --------------------------------------------------------------------------------

SpPsuKG   LLRESKGKSLNTNIDLVLNNAEKASLIAKELAVLKEKSSFFPTNTGNTFETKPVKQDFFYGKVSDKGVSSSKKKITETTS   388
DrPsuKG   VSELTKGKSLQANIALIRNNARVGSQIAHALSKLNENKEGHVRG-------------NIKTQKTM--DQK----------   347
DmPsuKG   IAKITEGRSLKSNIALIKNNAKVAAQIAASLCDVSTKLE-SPVQ----------------------------------KA   341
CePsuKG   VNELTQGASMATNIALLENNASIAGRLAAKLCDRRPLTI-------------------SQSQPTA--STT----------   330
EcPsuG    VAELTGGDSLKSNIQLVFNNAILASEIAKE--------------------------------------------------   306
AtPUMY    VNELTGGTSLAANIALVKNNALIGSQIAVALSQLM---------------------------------------------   330
AtPUKI    --------------------------------------------------------------------------------
EcPsuK    ------------------------------------------------------------------------------MR   2
```

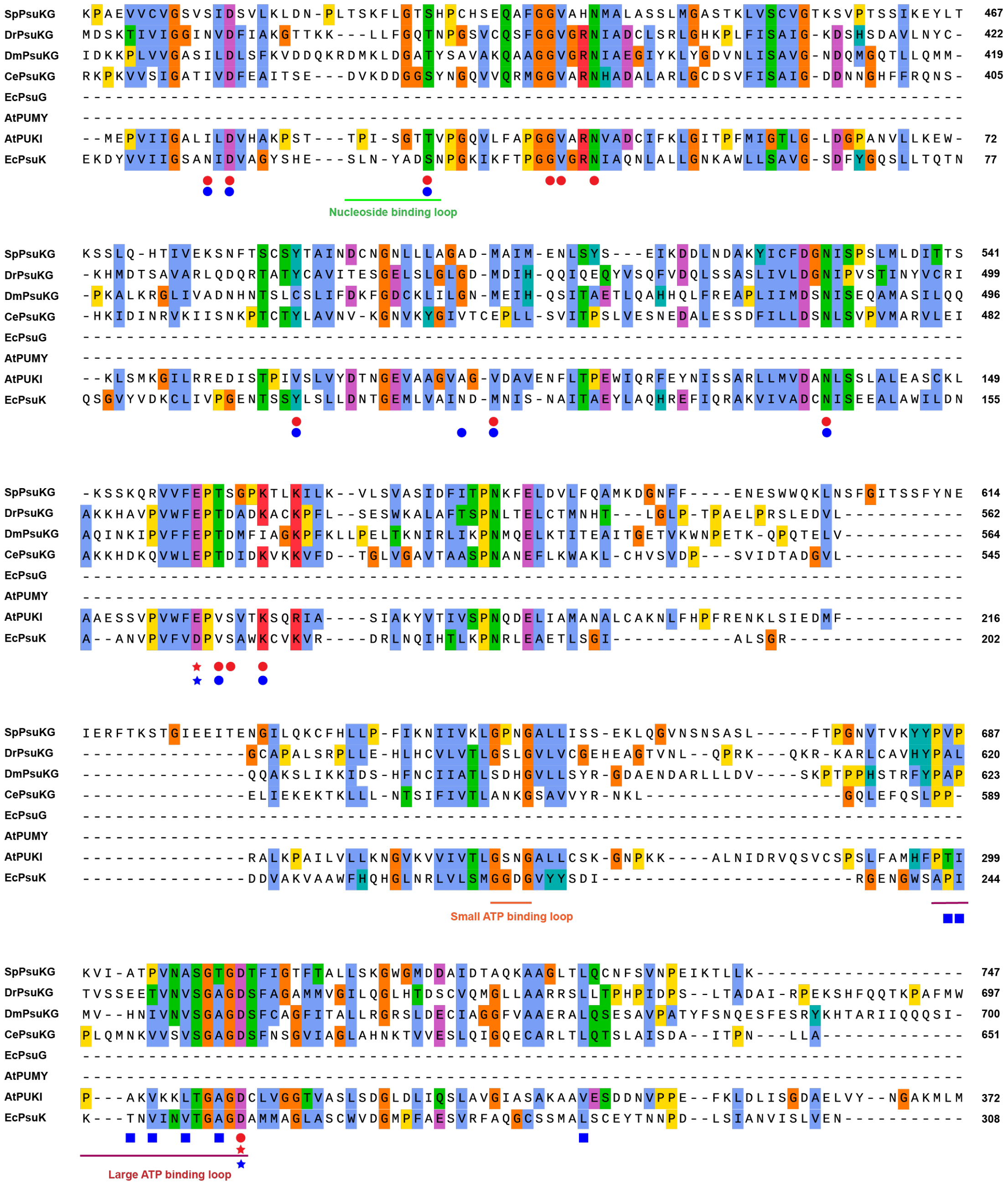

Supplementary Figure 4 Sequence alignment of PsuK and PsuG in different species. Structure-based sequence alignment of PsuK and PsuG with their homolog proteins. The residues involved in substrate binding are indicated by red (*Ec*PsuK) or blue (*At*PUKI) circles. The residues involved in ATP binding are indicated by blue squares for *At*PUKI. The asterisks indicated the potential residues for the catalytic reaction.

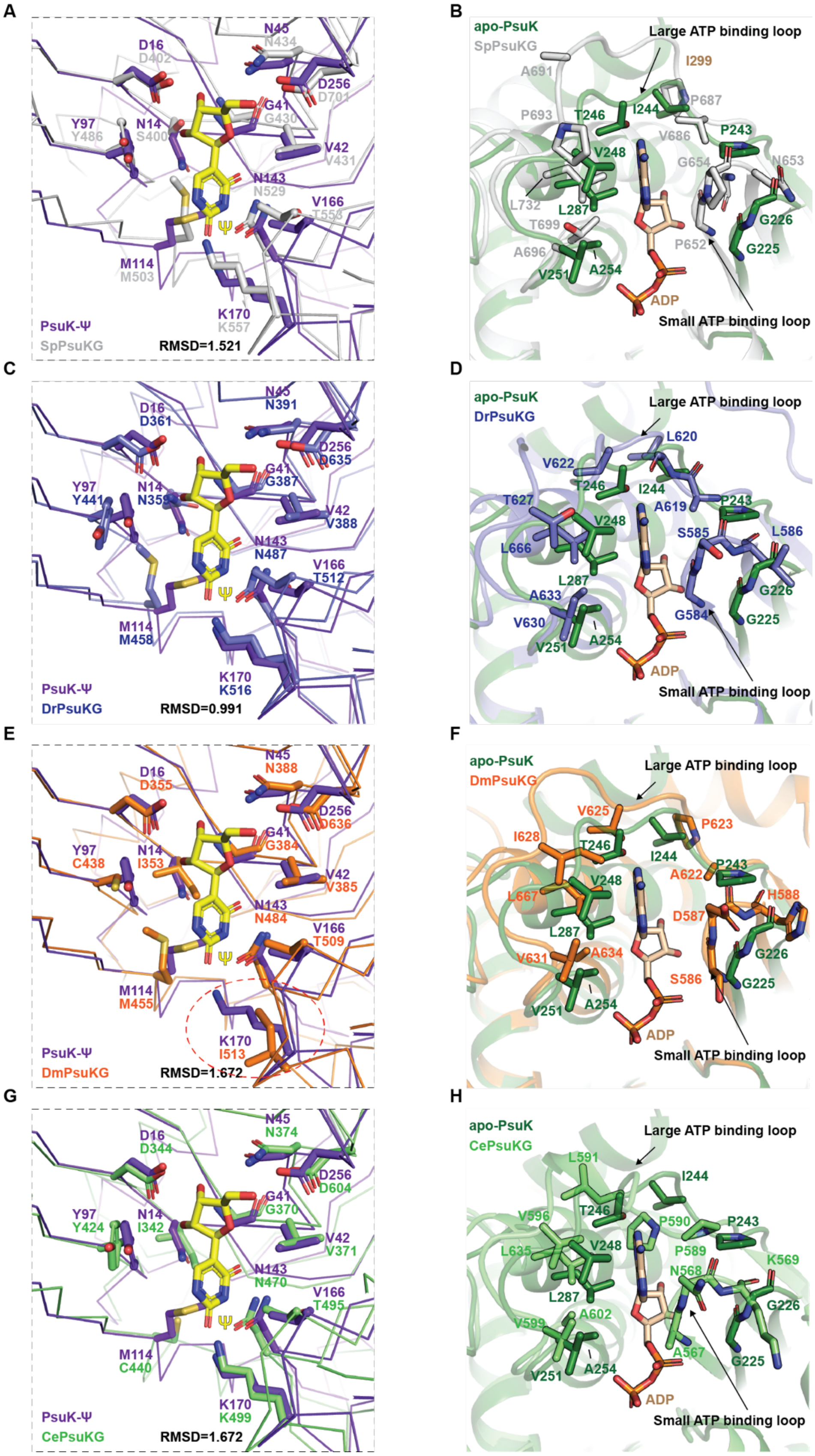

Supplementary Figure 5 Structure-based comparisons of *Ec*PsuK with the homolog proteins from different species. (**A**) Superposition of the substrate-binding pocket between PsuK and *S. pombe* PsuKG. (**B**) Comparisons of the ATP binding loops between *Ec*PsuK and

S. pombe PsuKG. (**C**) Superposition of the substrate-binding pocket between *Ec*PsuK and *D. rerio* PsuKG. (**D**) Superposition of the ATP binding loops between *Ec*PsuK and *D. rerio* PsuKG. (**E**) Superposition of the substrate-binding pocket between *Ec*PsuK and *D. melanogaster* PsuKG. The different residues of Lys170 in *Ec*PsuK and Ile353 in *D. melanogaster* are indicated by red dashed lines. (**F**) Superposition of the ATP binding loops between *Ec*PsuK and *D. melanogaster* PsuKG. (**G**) Superposition of the substrate-binding pocket between *Ec*PsuK and *C. elegans* PsuKG. (**H**) Superposition of the ATP binding loops between *Ec*PsuK and *C. elegans* PsuKG.

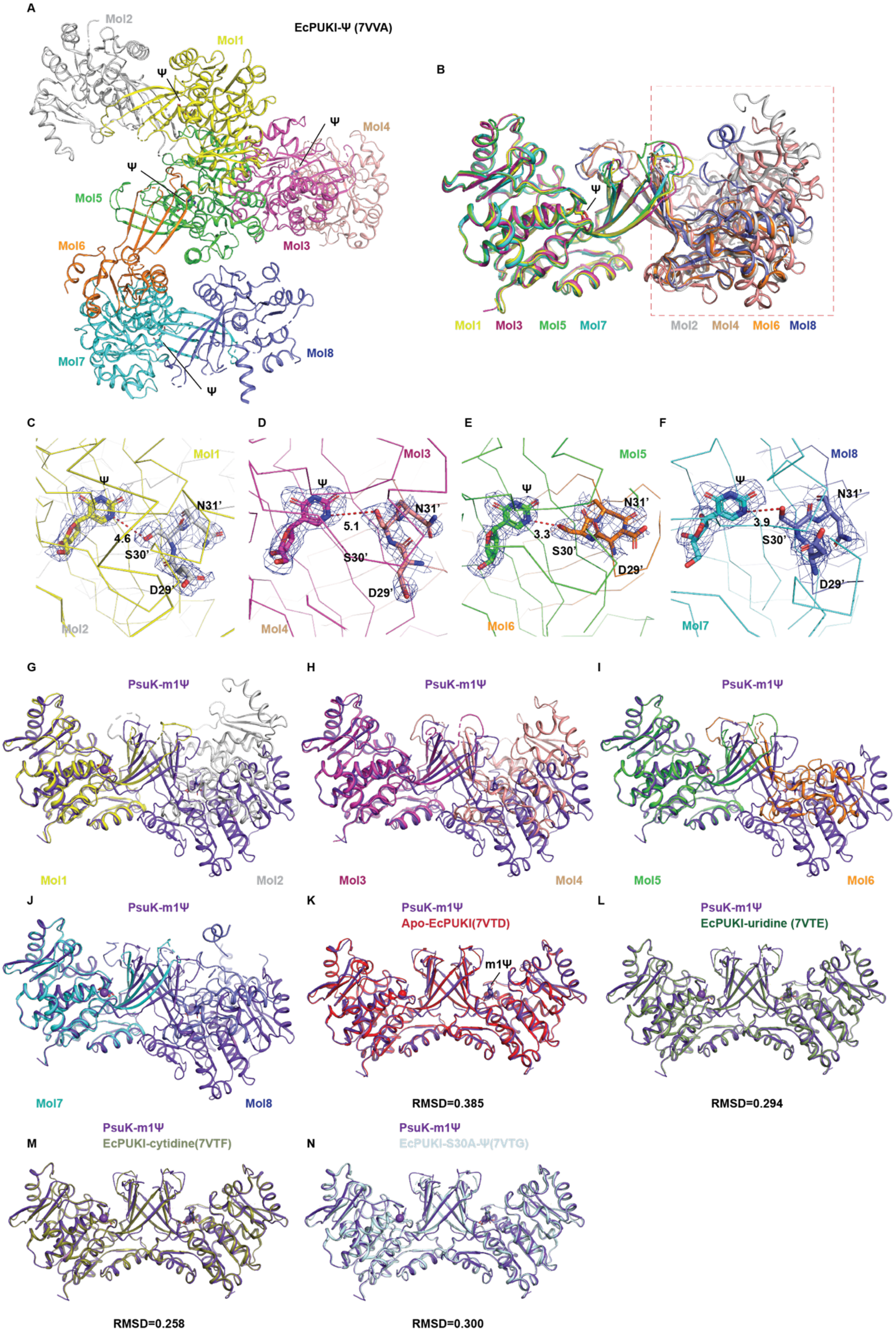
A
EcPUKI-Ψ (7VVA)
Mol1
Mol2
Mol3
Mol4
Mol5
Mol6
Mol7
Mol8
Ψ
B
Ψ
Mol1 Mol3 Mol5 Mol7
Mol2 Mol4 Mol6 Mol8
C
Mol1
Ψ
N31'
4.6
S30'
D29'
Mol2
D
Mol3
Ψ
5.1
N31'
S30'
D29'
Mol4
E
Mol5
Ψ
N31'
3.3
S30'
D29'
Mol6
F
Mol8
Ψ
N31'
3.9
S30'
D29'
Mol7
G
PsuK-m1Ψ
Mol1
Mol2
H
PsuK-m1Ψ
Mol3
Mol4
I
PsuK-m1Ψ
Mol5
Mol6
J
PsuK-m1Ψ
Mol7
Mol8
K
PsuK-m1Ψ
Apo-EcPUKI(7VTD)
m1Ψ
RMSD=0.385
L
PsuK-m1Ψ
EcPUKI-uridine (7VTE)
RMSD=0.294
M
PsuK-m1Ψ
EcPUKI-cytidine(7VTF)
RMSD=0.258
N
PsuK-m1Ψ
EcPUKI-S30A-Ψ(7VTG)
RMSD=0.300

Supplementary Figure 6 Structure of *Ec*PUKI homodimer undertake a series of conformational changes through sensing the Ψ substrate by Ser30'. (**A**) Structure of *Ec*PUKI-Ψ complex in the asymmetric unit (PDB code: 7VVA). The eight molecules are shown in different colors. (**B**) Superposition of the four homo-dimeric structures of *Ec*PUKI-Ψ complex in the asymmetric unit. The inactive parts without Ψ substrates are indicated by red dashed line. (**C-F**) The distance between the Ser30' and N^1 position of Ψ in the four dimer structures of *Ec*PUKI. The 2|Fo|–|Fc| σ-weighted map is contoured at 1.5σ. (**G-J**) Structure comparison of *Ec*PsuK-m1Ψ complex with the four dimer structures observed in *Ec*PUKI-Ψ complex. (**K**) Structure comparison of *Ec*PsuK-m1Ψ complex with the dimer structure observed in the apo-*Ec*PUKI structure (PDB code: 7VTD). (**L**) Structure comparison of *Ec*PsuK-m1Ψ complex with the structure of *Ec*PUKI-uridine complex (PDB code: 7VTE). (**M**) Structure comparison of *Ec*PsuK-m1Ψ complex with the structure of *Ec*PUKI-cytidine complex (PDB code: 7VTF). (**N**) Structure comparison of *Ec*PsuK-m1Ψ complex with the structure of *Ec*PUKI (S30A)-Ψ complex (PDB code: 7VTG).
